# HIV-1 subtype B- and F1-infected subjects display higher cross-clade T-Cell response than subtype C-infected subjects

**DOI:** 10.1186/1742-4690-9-S2-P150

**Published:** 2012-09-13

**Authors:** FH Côrtes, G Bello, C Vorsatz, JH Pilotto, B Grinsztejn, VG Veloso, AR Pinto, MG Morgado

**Affiliations:** 1 Oswaldo Cruz Institute/FIOCRUZ, Rio de Janeiro, Brazil; 2 Evandro Chagas Clinical Research Institute/FIOCRUZ, Rio de Janeiro, Brazil; 3 Nova Iguaçu General Hospital, Nova Iguaçu, Brazil; 4 Evandro Chagas Clinical Research Institute/FIOCRUZ, , Rio de Janeiro, Brazil; 5 Department of Microbiology, Immunology and Parasitology/UFSC, Santa Catarina, Brazil

## Background

The impact of the extensive genetic diversity of the HIV-1 group M isolates and its implications for vaccine design have long been discussed. Studies indicate that Gag and Nef conserved epitopes are commonly recognized and give rise to high cross-clade responses. The aim of this study was to compare T-cell responses to peptide pools derivate from subtype B, C and F1 consensus, among Brazilian subjects infected with those three HIV-1 subtypes.

## Methods

The study included 32 subjects infected with HIV-1 subtypes B (n=13), C (n=11) and F1 (n=8). Gag and Nef-specific T cell responses were evaluated by IFN-γ ELISpot assay, using peptide pools based on subtype B, C and F1 Brazilians consensus.

## Results

A high cross-clade response between subtypes B and F1 for both Gag and Nef regions was observed among HIV-1 subtype B- and F1-infected subjects. We also found no significant difference in magnitude of responses between subtype B and C consensus peptides in subtype B-infected subjects.In contrast, the magnitude of T cell responses to consensus C peptides in Gag region was significantly higher than to consensus B peptides among HIV-1 subtype C-infected subjects. In Nef, subtype C-infected subjects showed higher T cell responses to C than to F1 consensus peptides. Moreover, subtype F1-infected subjects presented lower responses to subtype C peptides than to subtype F1 and B ones.

## Conclusion

Overall, the level of cross-clade response between subtypes B and F1 was higher than between subtype C and B or between subtype C and F1. Our data suggest that significance of the HIV-1 group M genetic diversity for vaccine design may be dependent of the subtypes involved.

